# Oxidative DNA Damage and Cisplatin Neurotoxicity Is Exacerbated by Inhibition of OGG1 Glycosylase Activity and APE1 Endonuclease Activity in Sensory Neurons

**DOI:** 10.3390/ijms23031909

**Published:** 2022-02-08

**Authors:** Adib Behrouzi, Hanyu Xia, Eric L. Thompson, Mark R. Kelley, Jill C. Fehrenbacher

**Affiliations:** 1 Department of Pharmacology and Toxicology, Indiana University School of Medicine, Indianapolis, IN 46202, USA; abehrouz@iu.edu (A.B.); xiah@iu.edu (H.X.); elthomps@iupui.edu (E.L.T.); mkelley@iu.edu (M.R.K.); 2 Department of Pediatrics, Indiana University Simon Comprehensive Cancer Center, Indiana University School of Medicine, Indianapolis, IN 46202, USA; 3 Department of Pharmacology and Toxicology, Stark Neuroscience Research Institute, Indiana University Simon Comprehensive Cancer Center, Indiana University School of Medicine, Indianapolis, IN 46202, USA

**Keywords:** cisplatin, DNA damage, oxidative stress, chemotherapy-induced peripheral neuropathy, sensory neuron, neuropeptide, neurite outgrowth, base excision repair

## Abstract

Cisplatin can induce peripheral neuropathy, which is a common complication of anti-cancer treatment and negatively impacts cancer survivors during and after completion of treatment; therefore, the mechanisms by which cisplatin alters sensory neuronal function to elicit neuropathy are the subject of much investigation. Our previous work suggests that the DNA repair activity of APE1/Ref-1, the rate-limiting enzyme of the base excision repair (BER) pathway, is critical for neuroprotection against cisplatin. A specific role for 8-oxoguanine DNA glycosylase-1 (OGG1), the glycosylase that removes the most common oxidative DNA lesion, and putative coordination of OGG1 with APE1/Ref-1 in sensory neurons, has not been investigated. We investigated whether inhibiting OGG1 glycosylase activity with the small molecule inhibitor, TH5487, and/or APE1/Ref-1 endonuclease activity with APE Repair Inhibitor III would alter the neurotoxic effects of cisplatin in sensory neuronal cultures. Sensory neuron function was assessed by calcitonin gene-related peptide (CGRP) release, as a marker of sensitivity and by neurite outgrowth. Cisplatin altered neuropeptide release in an inverse U-shaped fashion, with low concentrations enhancing and higher concentrations diminishing CGRP release. Pretreatment with BER inhibitors exacerbated the functional effects of cisplatin and enhanced 8oxo-dG and adduct lesions in the presence of cisplatin. Our studies demonstrate that inhibition of OGG1 and APE1 endonuclease activity enhances oxidative DNA damage and exacerbates neurotoxicity, thus limiting oxidative DNA damage in sensory neurons that might alleviate cisplatin-induced neuropathy.

## 1. Introduction

Chemotherapy-induced peripheral neuropathy (CIPN) is a very common ailment of cancer patients and survivors [1] and is defined by the presence of one or more of the following symptoms starting in the hands and feet: dysesthesia, numbness, sharp stabbing or burning pain, hypersensitivity to cold, and loss of reflexes. Cisplatin is a chemotherapeutic commonly used for the treatment of many cancers, including testicular, bladder, ovarian, head and neck, lung, and cervical cancers [2], and causes some of the most severe neuropathy. This neuropathy can result in patients withdrawing from their treatment schedules and disrupting the treatment of their cancer.

The mechanisms by which cisplatin alters sensory neuronal function to result in neuropathy are the subject of much investigation. Preclinical studies have suggested that mitochondrial toxicity [3,4,5], changes in calcium handling [6], oxidative stress [6,7], DNA damage [7,8,9], and morphological changes [9,10] all occur in sensory neurons secondary to cisplatin exposure. Cisplatin contains a central platinum ion, which allows for the drug to cross-link DNA and thereby inhibit cellular replication [11] in cancer cells. In addition to DNA adducts induced by cisplatin, the drug also increases oxidative stress with increases in reactive oxygen species (ROS) [12] and subsequent oxidative DNA damage [13,14] to further inhibit cellular replication. Even though sensory neurons are unaffected by the inhibition of replication due to their post-mitotic status, DNA damage can still affect function through changes in gene transcription [15] and intracellular signaling pathways activated in response to DNA damage and the cognate processes engaged to repair DNA lesions.

The base excision repair (BER) pathway is primarily responsible for repairing nonbulky DNA damage, such as oxidative and alkylation DNA damage. Because of the low oxidation potential for guanines, the most prevalent DNA lesion induced by reactive oxygen species is 7,8-dihydro-8-oxo-2′-deoxyguanosine (8oxo-dG) [16,17,18]. The first step of BER is to recognize and remove the damaged base, 8-oxodG, and is mediated by 8-oxoguanine DNA glycosylase-1 (OGG1) [19]. Removal of the base generates an apurinic/apyrimidinic (AP) site, which is then recognized and processed by the multifunctional AP endonuclease 1/Ref-1 (APE1/Ref-1), which cleaves the DNA backbone, allowing DNA polymerase β (Polβ) to insert the missing base through template-directed synthesis [20]. We previously demonstrated that APE1/Ref-1 knockdown exacerbates the neuronal effects of cisplatin and that overexpression of APE1/Ref-1 rescues the neurons [7,21,22], suggesting that oxidative DNA damage mediates, at least in part, the deleterious effects of cisplatin on neuronal function. Oxidative DNA lesions, such as 8oxo-dG, near cisplatin-DNA cross links might also interrupt cross-link lesion repair, increasing any impact of the oxidative damage alone [23].

What is still unknown are the mechanisms by which oxidative DNA damage alters neuronal function. Work in cancer cells has demonstrated that the presence of oxidative DNA lesions alone can alter gene transcription [24,25,26,27]; however, additional work has shown that the binding of enzymes critical for BER can also elicit changes in transcription that could have variable effects on gene transcription [28,29]. Indeed, inhibition of OGG1 glycosylase activity by the small molecule, TH5487, prevents inflammatory gene expression changes induced by cellular exposure to TNFα [28].

In these studies, we expand on our previous findings to identify the relative roles of the major glycosylase required for 8oxo-dG lesion removal, OGG1, and APE1/Ref-1 on mitigating the damage to sensory neurons induced by cisplatin. We determined that inhibiting the activity of either OGG1 or APE1/Ref-1 increases 8-oxodG levels in neuronal cultures and that this increase is exacerbated by cisplatin. Functionally, cisplatin has variable effects on sensory neuron function, with increases in neuropeptide release at low concentrations, and a diminution of release at higher concentrations. Inhibition of the BER enzymes appears to exacerbate the effects of cisplatin to facilitate the desensitization of neuropeptide release from sensory neurons. These data support a role for oxidative DNA damage, and specifically 8oxo-dG formation, as a mediator of cisplatin-induced neurotoxicity.

## 2. Results

### 2.1. Inhibition of Either OGG1 Glycosylase Activity or APE1 DNA Repair Activity Diminishes the Sensitizing Effects of Cisplatin on Neuropeptide Release

Cellular exposure to cisplatin causes the formation of platin-DNA crosslinks and the generation of ROS [22,30,31,32], producing oxidative DNA damage that is primarily repaired by the BER pathway [33]. Left unrepaired, oxidative DNA damage contributes to neuronal toxicity induced by cisplatin, as determined by experiments in which APE1 expression or activity was modulated [7,21,22]. The desire to more completely understand the base excision pathway and the contribution of each step of the pathway to alterations in neuronal function led us to examine the effects of inhibiting OGG1 glycosylase activity and/or APE1 DNA repair activity.

**Figure 1:**
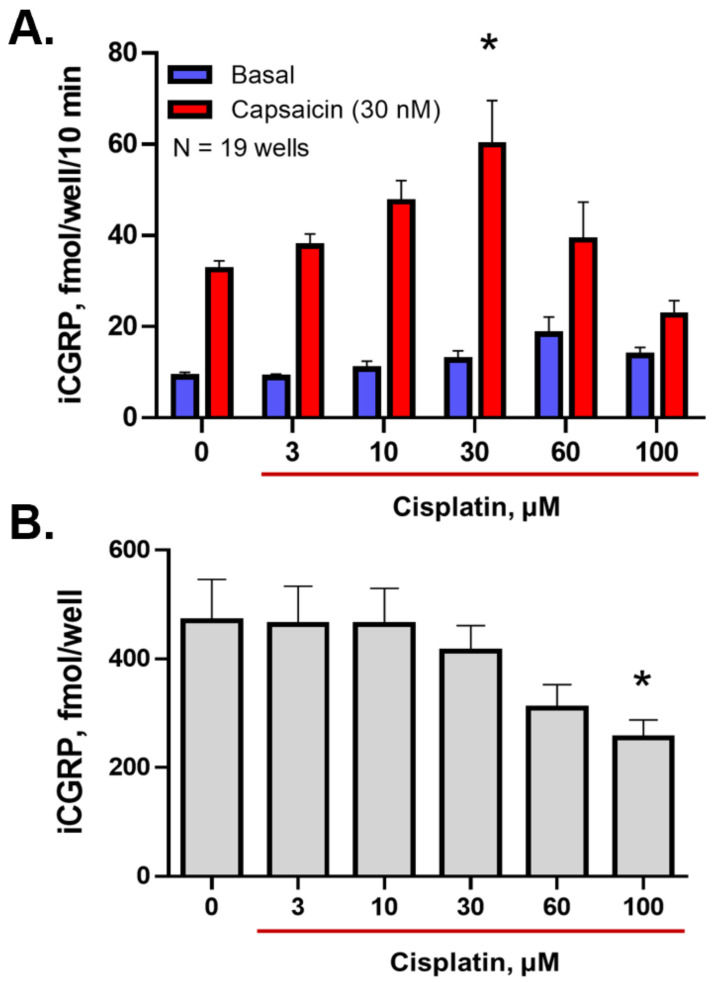
CGRP release from neuronal cultures is altered following exposure to cisplatin. (**A**). Columns represent the mean ± SEM of CGRP release stimulated by a 10 min exposure to 30 nM capsaicin following a 24 h exposure to increasing concentrations of cisplatin (30 μM). * *p* < 0.0001 comparing stimulated release in CIS to no treatment control, two-way ANOVA with Tukey’s posttest. (**B**). Each column represents the mean ± SEM of the total CGRP content following exposure to cisplatin treatments. * *p* < 0.05 comparing content in CIS (100 μM) to no treatment control, one-way ANOVA with Dunnett’s posttest.

Before we could examine the effects of BER inhibitors, we needed to establish the effects of increasing concentrations of cisplatin on CGRP release and content, as cisplatin and other chemotherapeutics have demonstrated an inverse U-shaped effect on release, with low concentrations of the drug enhancing the stimulated release of the peptide and higher concentrations diminishing the release of the peptide [34]. Therefore, we treated neuronal cultures with increasing concentrations of cisplatin, using dd water as a vehicle, for 24 h prior to a release experiment. As illustrated in Figure 1A, cisplatin significantly enhanced the stimulated release of CGRP at a concentration of 30 μM from 32.4 ± 2.0 to 59.8 ± 9.7 fmol/well/10 min; however, increasing the concentration of cisplatin further resulted in a drop in the stimulated release of CGRP. Figure 1B demonstrates that treated sensory neuron cultures with high concentrations of cisplatin (100 μM) diminishes the neuropeptide content from 469.8 ± 76.6 to 255.3 ± 32.37 fmol/well; however, no significant effect of a 30 μM cisplatin treatment on CGRP content was observed. 

**Figure 2:**
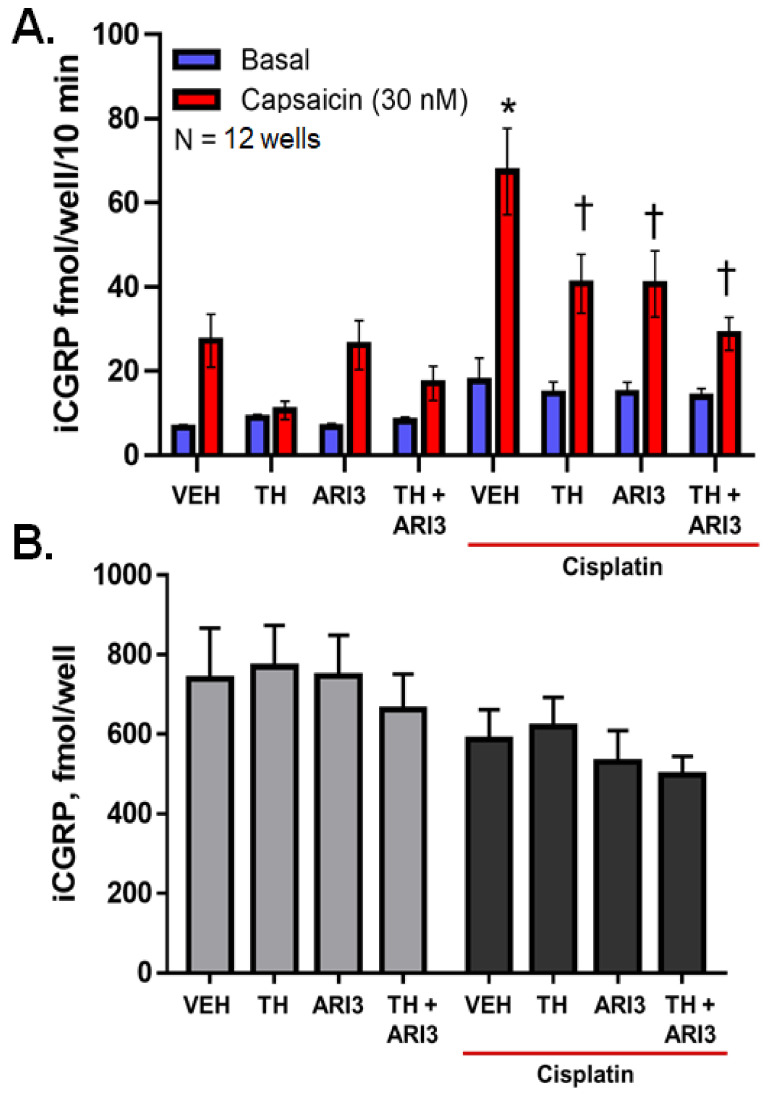
CGRP release from neuronal cultures is altered following exposure to cisplatin and the changes in stimulated CGRP release following exposure to base excision repair inhibitors. (**A**). Columns represent the mean ± SEM of CGRP release stimulated by a 10 min exposure to 30 nM capsaicin following a 24 h exposure to cisplatin (30 μM). * *p* < 0.0001 comparing stimulated release in CIS to VEH controls; † *p* < 0.001 comparing CIS effects in the presence and absence of base excision repair inhibitors, two-way ANOVA with Tukey’s posttest. (**B**). Each column represents the mean ± SEM of the total CGRP content following the indicated treatments.

To examine how inhibition of OGG1 and APE1 alters the effects of cisplatin, we exposed neuronal cultures to TH5487 (3 μM), ARI3 (10 μM), a combination of the drugs, or to vehicle controls for 48 h and then added vehicle or cisplatin (30 μM) for the last 24 h in culture prior to the experiment examining basal- and capsaicin-stimulated release of the putative nociceptive neuropeptide, CGRP. As illustrated in Figure 2A, exposure to capsaicin (bars in red) stimulates the release of CGRP. Exposure of the cultures to cisplatin elicits a sensitization of that stimulated release from an average of 27.2 ± 6.3 to 67.4 ± 10.3 fmol/well/10 min. Pretreatment with either BER inhibitor alone attenuated the increase in release elicited by cisplatin and combining the inhibitors trended towards an additive effect; however, the effects of combination were not significant. The changes in release of CGRP were not secondary to an altered content of CGRP in the neurons, as the total content of CGRP was similar in cultures treated with BER inhibitors in the absence and presence of cisplatin (Figure 2B).

### 2.2. Inhibition of Either OGG1 Glycosylase Activity or APE1 DNA Repair Activity Enhances Basal Levels of 8oxo-dG Lesions and This Effect Is Exacerbated by Cisplatin Treatment

**Figure 3:**
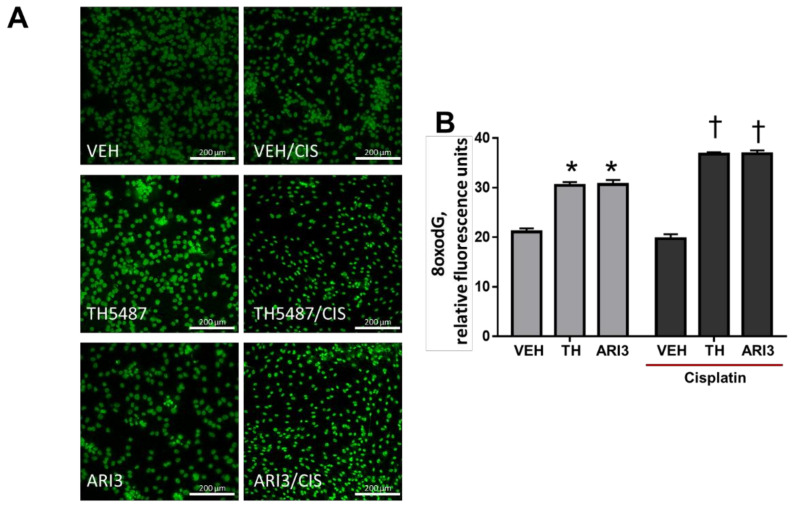
Effects of cisplatin and base excision repair inhibitors on 8-oxodG levels in cells adjacent to sensory neurons. (**A**), Representative immunostaining for 8-oxodG (green) in sensory neuron cultures. The original magnification was ×20. Scale bar represents 200 µm. (**B**), Quantitative analysis of the integrated density of 8-oxodG staining within PGP9.5+ regions of the image field, acquired as relative fluorescence units using Cytation5 software. * *p* < 0.0001 comparing TH and ARI3 to VEH; † *p* < 0.0001 comparing TH/CIS and ARI3/CIS to inhibitors alone, two-way ANOVA with Tukey’s posttest.

The importance of DNA repair pathways to maintain the integrity of DNA in post-mitotic cells, such as neurons, has been highlighted recently. The platin drugs have the potential to create oxidative lesions and adducts within DNA and RNA and the base and nucleotide excision repair pathways, respectively, are thought to eliminate that damage. We investigated the effects of a 48 h pretreatment with BER inhibitors on 8oxo-dG levels in neuronal cultures in the absence and presence of a 6 h exposure to cisplatin. The images in Figure 3A depict representative micrographs of 8oxo-dG immunoreactivity in sensory neuron cultures. To limit the 8oxo-dG signal to sensory neurons, we utilized a PGP9.5 mask during the image analysis, so that only those nuclei in close proximity to sensory neurons were assessed. The quantification of 8oxo-dG is depicted in Figure 3B. As we expected, inhibiting OGG1 or APE1 resulted in an increase in 8oxo-dG levels from a basal level of 21.1 ± 0.7 to 30.4 ± 0.7 and 30.7 ± 0.9 relative fluorescence units (RFU), respectively, even in the absence of cisplatin treatment, suggesting that there is a basal level of oxidative damage within the neurons that is constantly being repaired via the enzymes in the BER pathway. Surprisingly, we did not observe an increase in 8oxo-dG levels in cultures treated with cisplatin; however, we did see an exacerbation of damage in the inhibitor-treated cells treated with cisplatin, as 8oxo-dG levels increased to 36.8 ± 0.4 and 36.9 ± 0.7 RFU in the presence of TH and ARI3, respectively, compared with treatment in the absence of cisplatin. 

### 2.3. Inhibition of APE1 DNA Repair Activity Enhances Cisplatin Adduct Levels in Neuronal Cultures

**Figure 4:**
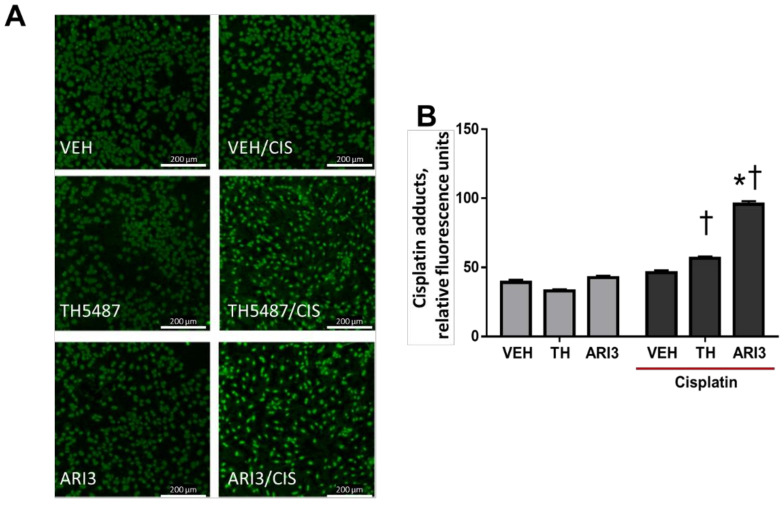
Effects of cisplatin and base excision repair inhibitors on cisplatin adduct levels in cells adjacent to sensory neurons. (**A**) Representative immunostaining for cisplatin adducts (green) in sensory neuron cultures. The original magnification was ×20. Scale bar represents 200 µm. (**B**) Quantitative analysis of the integrated density of adduct staining within PGP9.5+ regions of the image field, acquired as relative fluorescence units using Cytation5 software. † *p* < 0.0001 comparing TH/CIS and ARI3/CIS to TH/VEH and ARI3/VEH, respectively, * *p* < 0.0001 comparing VEH/CIS to ARI3/CIS; two-way ANOVA with Tukey’s posttest.

Although the focus of these studies is on oxidative DNA damage and the base excision repair pathway, the most recognized neuronal DNA damage elicited by cisplatin is that of adduct formation [35,36]. We previously demonstrated that diminishing the expression of APE1 increased the presence of cisplatin adducts, suggesting possible crosstalk between APE1 and the NER pathway [37]. To ascertain whether pharmacological inhibition of OGG1 or APE1 alters levels of cisplatin adducts, we pretreated neuronal cultures with BER inhibitors for 48 h in the absence and presence of a 6 h exposure to cisplatin and then measured adducts. The images in Figure 4A depict representative micrographs of cisplatin adduct immunoreactivity in sensory neuron cultures. To limit the adduct signal to sensory neurons, we utilized a PGP9.5 mask during the image analysis, so that only those nuclei in close proximity to sensory neurons were assessed. The quantification of cisplatin adducts is depicted in Figure 4B. There was some background staining for cisplatin adducts in all conditions; however, we did not observe an expected increase in cisplatin adducts in the absence of BER inhibitors. We did see a significant increase in cisplatin adducts with treatment of either of the BER inhibitors compared with background levels in vehicle-treated cultures. Furthermore, inhibition of APE1 dramatically increased the levels of cisplatin adducts from a background level of 46.2 ± 1.7 with cisplatin alone to 95.7 ± 2.3 RFU with ARI3 pretreatment combined with cisplatin treatment.

### 2.4. Cisplatin Elicits a Decrease in Neurite Outgrowth in Cultures of Sensory Neurons, and This Is Partially Attenuated by Inhibition of OGG1 Glycosylase Activity

**Figure 5:**
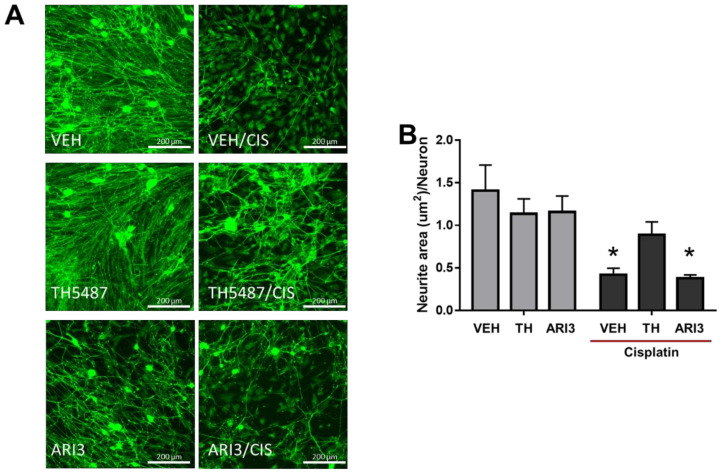
Effects of cisplatin and base excision repair inhibitors on neurite outgrowth. (**A**) Representative immunostaining for PGP9.5 (green) in sensory neuron cultures. The original magnification was ×20. Scale bar represents 200 µm. (**B**) Quantitative analysis of the axonal area of PGP9.5 staining, acquired using Cytation5 software. * *p* < 0.01 comparing the indicated groups to the Vehicle-treated control, two-way ANOVA with Tukey’s posttest.

Chemotherapeutics are known to induce a loss of epidermal innervation in cancer patients [38], and this effect has been recapitulated in vitro, where the exposure of sensory neuronal cultures to various different chemotherapeutics induces a loss of neurite length and/or branching [39,40,41]. To determine whether inhibition of the base excision repair pathway alters the effects of cisplatin to diminish neurite outgrowth, we exposed neuronal cultures to TH5487 (3 μM), ARI3 (10 μM), or to vehicle controls for 48 h and then added vehicle or cisplatin (30 μM) for the last 24 h in culture prior to fixation of the cells to assess neurite areas. As pictured in Figure 4A, the treatment with BER inhibitors alone did not alter neurite outgrowth. As we expected, cisplatin diminished the neurite area per neuron captured from 1.4 ± 0.3 in the presence of vehicle to 0.4 ± 0.1 μm^2^ following a 24 h cisplatin treatment (Figure 4B). Interestingly, we saw that pretreatment with the OGG1 inhibitor partially attenuate the loss of neurite area in the presence of cisplatin treatment to 0.9 ± 0.1 μm^2^; however, there was no effect of inhibition of APE1-mediated DNA repair on the neurite area following cisplatin treatment (Figure 5).

## 3. Discussion

It is well established that treating animals with cisplatin results in the production of platinum adducts in sensory neurons [35,36] and levels of adduct formation have been correlated with the degree of toxicity to the peripheral sensory neurons [8,42]. The nucleotide excision repair (NER) pathway catalyzes the removal of cisplatin adducts from nuclear DNA and actively repairs nuclear adducts in sensory neurons [37,43]. Indeed, our data suggest a very robust clearance of adducts following exposure to cisplatin, as we did not observe significant adduct accumulation in the absence of DNA repair inhibitors. Furthermore, investigational drugs that increase the clearance of nuclear cisplatin adducts in sensory neurons diminish the neurotoxicity induced by cisplatin [9].

In addition to alterations in nuclear transcription through the formation of cisplatin adducts within nuclear DNA, cisplatin can also form adducts within the mitochondrial DNA (mtDNA). As NER is thought to be ineffective or minimal in mitochondria [44,45], adduct damage in the mitochondria of sensory neurons can have long-term effects on mitochondrial function. Podratz and colleagues [4] demonstrated a correlation between cisplatin-induced decreases in mtDNA replication and the diminished transcription of mitochondrial transcripts. Aberrant expression of mitochondrial transcripts could contribute to deficiencies of the neuronal mitochondria following exposure of the neurons to cisplatin. Alternatively, mitochondrial DNA damage could serve to recruit p53 to the mitochondria, which has been shown to alter mitochondrial bioenergetics and contribute to ROS generation and oxidative DNA damage [9,46,47,48].

Cisplatin-DNA adducts are not the only type of DNA lesions produced by neuronal exposure to cisplatin. Oxidative damage is also induced by cisplatin [13,48,49], resulting in the formation of 8-oxodG lesions in DNA and RNA. Inhibition of either OGG1 glycosylase activity or APE1 DNA repair activity resulted in an increase in 8-oxodG lesions in sensory neuron cultures, supporting the notion that the BER pathway is very active. In fact, we did not observe an increase in 8-oxodG lesions induced by cisplatin, unless we inhibited the BER pathway, suggesting that the cells are constantly repairing the damage induced by cisplatin. Although we were focused on DNA damage repair, the antibody that we use to detect 8-oxodG lesions likely recognizes both DNA and RNA 8-oxodG. Therefore, we included RNase A to limit our findings, specifically to 8-oxodG lesions on DNA. It is unclear, however, what impact the DNA lesions themselves or the repair of the lesions might have on sensory neuron function. Oxidative DNA damage was thought to disrupt transcription by preventing transcription factor binding to consensus sequences within gene promoter elements [24,25,26] and by stalling the progress of RNA polymerase II during transcription [27]. Transcriptional repression secondary to oxidative damage, however, has been shown to be dependent upon an abasic site produced by OGG1-induced lysis of 8-oxodG, suggesting that BER activity is essential to observe effects of oxidative lesions [50]. In an opposite role, OGG1 bound to 8-oxodG has been reported to serve as an epigenetic mark to enhance gene transcription secondary to inflammatory stimuli by increasing the recruitment of transcription factors to promoter elements of genes [28,51]. Furthermore, the bound 8-oxodG:OGG1 complex has been shown to have signal transduction functions, including Ras and Rho activation [52,53]. Advanced investigation into the mechanisms by which the BER enzymes enhance transcription has identified the formation of guanine-rich G-quadruplexes within the promoter sequences of genes differentially regulated by oxidative damage [43]. G-quadruplexes (G4) are secondary structures of guanine-rich nucleic acids that form a helical structure due to guanine: guanine interactions and are commonly seen in promoter regions, 5′ and 3′ untranslated regions, and within telomeric regions of DNA [54]. In addition to G-quadruplexes, oxidative DNA damage has also been shown to alter cruciform DNA sequences in the promoter elements of genes [55]. The formation of G4 and cruciform structures can enhance or repress the transcription of genes, depending on whether the secondary structure forms within the template or nontemplate strands [55,56], thus oxidation-induced increases in DNA secondary structures provide another means by which oxidative damage alters gene transcription. Recent reports suggest that APE1 binding to AP sites within G4 sequences increases the folding of DNA into G4 structures and maintenance of APE1 binding, via APE1 acetylation, stabilizes the G4 and increases transcription factor recruitment to the promoter [29]. The effects of APE1 are not limited to DNA, as recent developments demonstrate that APE1 is also involved in the repair of damaged RNA, particularly oxidatively damaged RNA [57,58,59]. A clear understanding of what precedes APE1 activity on ribose AP sites is unclear; however, we cannot exclude the possibility that cisplatin also enhances the formation of 8-oxodG lesions within RNA and that APE1 can play a role in repairing the oxidative damage in RNA.

Although we did not observe the presence of oxidative lesions 6 h after the start of cisplatin treatment, we did observe effects of cisplatin on neuronal function, which was altered by inhibitors of the BER pathway. The effects of the BER inhibitors could be interpreted in alternate ways, due to the inverse U-shaped cisplatin concentration curve and further experiments are needed to conclusively identify whether the inhibitors have a neuroprotective or neurotoxic effect. The effect of BER inhibitors to reduce the sensitizing effects of cisplatin could be interpreted as a true inhibition of the effects of cisplatin. Alternatively, the BER inhibitors might be creating a rightward shift in the cisplatin concentration curve so that, with the inhibitor treatments, the effects of cisplatin are exacerbated and produce desensitizing effects on release (Figure 1A and Figure 2A). We previously demonstrated that overexpressing endonuclease-competent APE1 or pharmacologically increasing APE1 DNA repair activity is neuroprotective against the effects of cisplatin in sensory neurons [7,22,60], which would support the latter interpretation: a role for the inhibitors to exacerbate cisplatin-induced neurotoxicity. However, the precise mechanisms by which oxidative DNA damage or the repair thereof alters neuronal function are still unclear. In order to determine which aspect of cisplatin-induced DNA damage leads to neurotoxicity, we used selective inhibition of either OGG1, APE1 or a combined inhibition of both BER enzymes. It is known that base excision repair enzymes work in concert to facilitate DNA repair: OGG1 has higher specificity for 8-oxodG [61], cleaves the lesioned base at a higher rate, and has a lower affinity for the abasic site to facilitate APE1 binding and endonuclease activity [62] when APE1 is present. Indeed, we observed equivalent effects of inhibiting either enzyme on both the presence of 8-oxodG lesions and the modulation of neuropeptide release, suggesting that OGG1 activity to remove a lesioned base is dependent upon APE1 activity in sensory neuron cultures. Combined inhibition of OGG1 and APE1 did not have a significant impact on CGRP release compared with either drug alone, suggesting that neither enzyme has a role outside of the BER pathway to alter neuronal function. This was a little surprising, as we demonstrated some crosstalk between APE1 and the NER DNA repair pathway, as inhibition of APE1 enhanced the levels of cisplatin adducts. These findings corroborate our previous findings, that cisplatin adducts were enhanced by knockdown of APE1/Ref-1 expression and diminished by overexpression of APE1/Ref-1 [37]. However, previous work by others has demonstrated a correlation between platin adduct formation and neurotoxicity effects [8,42]; therefore, we will continue to examine the role of APE1 in the NER pathway. Interestingly, the interactions between APE1 and the NER pathway may be cell-dependent, as APE1 did not promote adduct repair in some cancer cells [23,63]. An interaction of BER proteins with other DNA repair pathways has been observed [64]; for example, Polβ polymerase can recruit mismatch repair (MMR) proteins [65] and the BER proteins, APE1 and PARP1, interact directly with Cockayne Syndrome B protein, which is critical for transcription-coupled and global nucleotide excision repair [66]. In addition to endonuclease/lyase functions to repair DNA damage, APE1 binds to RNA and may contribute to phase separation to organize biocondensates, which could enhance BER [59]. These findings support the notion that APE1 may modulate the DNA damaging effects of cisplatin beyond the repair of oxidative DNA lesions, suggesting that manipulation of the BER pathway may have indirect consequences on other DNA repair pathways and vice versa. Because OGG1 and APE1 work in a linear pathway to repair 8-oxodG lesions, we do not anticipate an additive effect on the inhibitors to alter the presence of 8-oxodG. However, we will examine that possibility in future experiments. Similarly, we will assess the combination drug effect on adduct formation, since OGG1 inhibition seemed to have a small effect on adduct levels in sensory neurons. 

In addition to changes in neuronal sensitivity induced by cisplatin, chemotherapeutics can also drive reductions in intraepidermal innervation of the skin (in vivo) and neurite length (in vitro) [39,40,41,67]. We first examined whether inhibiting the activity of either OGG1 and APE1 would alter neurite outgrowth, but saw no effect of either drug. Interestingly, we found that inhibition of OGG1 abrogated the effects of cisplatin to decrease neurite outgrowth. It is unclear how DNA damage or OGG1 intracellular signaling might alter outgrowth. Signaling by RhoA in sensory neurons has been shown to negatively regulate neurite outgrowth [68]. Cisplatin could enhance RhoA activation via engagement of OGG1:8-oxydG signaling to induce neurite retraction, and thus inhibition of OGG1 could reverse the effects of cisplatin to decrease outgrowth. Because of the rescue effect of OGG1 inhibition and apparent lack of effect of APE1 inhibition on neurite outgrowth, we did not test the effects of the drugs in combination. Further studies will be performed to investigate the effects of a RhoA inhibitor on cisplatin-induced changes in neurite outgrowth.

Recent reports suggest that cisplatin can elicit the transition of sensory neurons into a senescent-like state [69,70]. Senescence in post-mitotic neurons of the central nervous system can be driven by numerous cell stressors, including DNA damage and oxidative stress, and is understood to be a way to accommodate stress without eliciting apoptosis [71]. However, senescent cells are known to release cytokines and chemokines (senescence-associated secretory phenotype) [72], which could result in the sensitization of neighboring non-senescent cells. Indeed, in an animal model whereby senescent cells could be selectively ablated, cisplatin-induced neuropathy is alleviated [70]. APE1 deficiency in primary fibroblasts contributes to the development of senescence [73]. Future studies in our laboratory will elucidate the contribution of DNA damage to the initiation of cisplatin-induced senescence in sensory neurons and determine whether increasing APE1 DNA repair activity could prevent the onset of senescence. 

**Figure 6:**
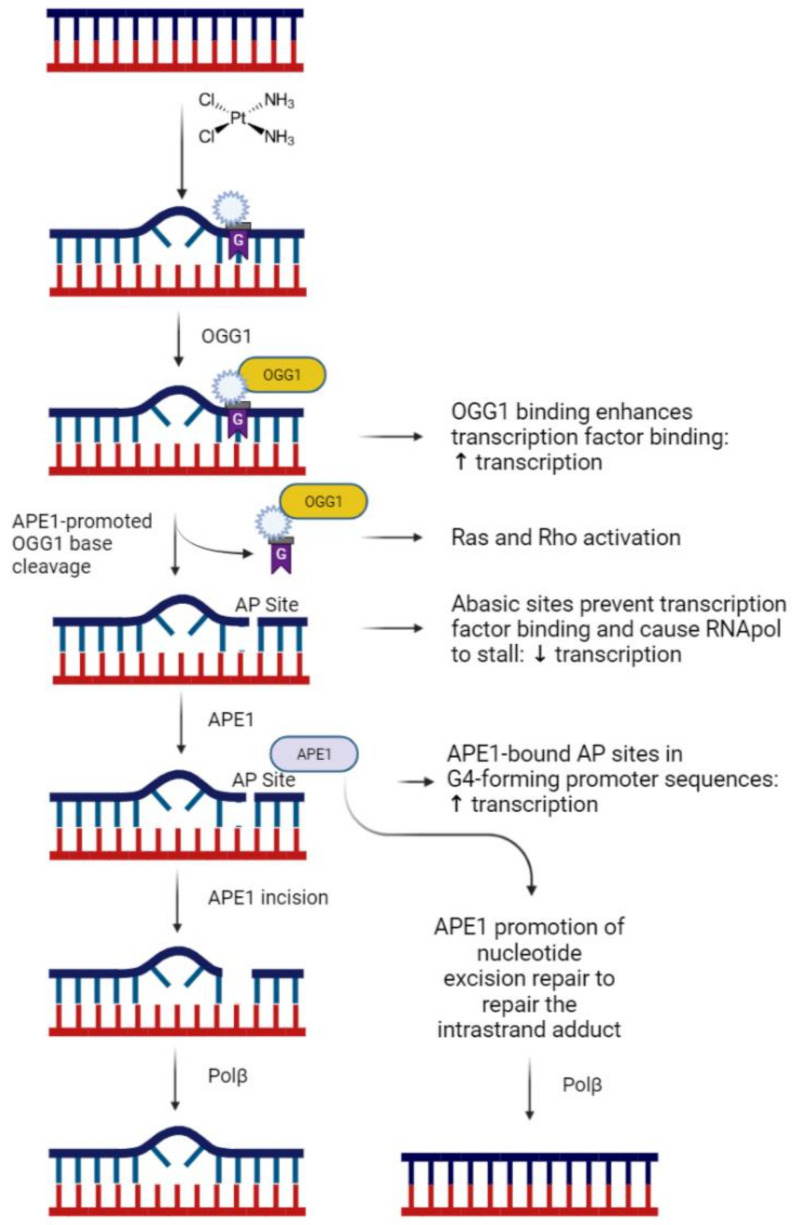
A summary of the proposed roles of oxidative DNA damage, OGG1 and APE1 in sensory neurons. Figure created with Biorender.com, accessed on 29 December 2021.

Based upon the above studies, we propose a model for cisplatin-induced neurotoxicity that is likely mediated by oxidative DNA damage (Figure 6), suggesting that the enhanced activation of OGG1 and APE1 endonuclease activity would ameliorate neurotoxicity. One limitation of our study is that we largely limited our examination to peptidergic sensory neurons. Further work will determine whether cisplatin induces DNA damage in all of the subpopulations of sensory neurons in a similar manner. Future studies will more precisely dissect the function of OGG1 and APE1 to examine how the activity of these proteins alters sensory neuron function.

## 4. Materials and Methods

### 4.1. Animals

Experiments were conducted according to protocols approved by the Institutional Animal Care and Use Committee at Indiana University School of Medicine (protocol #20098), Indianapolis, IN and in compliance with the National Institutes of Health Guide for the Care and Use of Laboratory Animals. Experiments were performed on primary cultures of sensory neurons derived from the DRG of adult male Sprague Dawley rats (150–250 g; Envigo, Indianapolis, IN, USA). Animals were housed in group cages in a light-controlled room with food and water available ad libitum.

### 4.2. Isolation of Primary Sensory Neuron Cultures

DRG were harvested and cultured as described previously [34]. Dissociated DRG cells were added to plates previously coated with poly-D-lysine and laminin [~15,000 cells/well in 24-well plates for CGRP release assays and neurite imaging experiments and ~3000 cells/well in 4-well chamber slides for the assessment of DNA damage]. Cells were grown in F-12 growth media supplemented with 10% heat-inactivated horse serum, 2 mM glutamine, and 50 µg/mL penicillin and streptomycin in the presence of 10 ng/mL NGF. Cells were maintained in an atmosphere of 3% CO_2_ at 37 °C, and media was changed every other day. Stocks (10 mM) of the OGG1 and APE1 inhibitors [28,74,75], TH5487 and ARI3, were prepared in N-Methyl-2-pyrrolidone and diluted in media to the appropriate concentrations (TH5487: 3 μM; ARI3: 10 μM) and exposed to the treatment wells for 48 h prior to release or fixation. For cisplatin treatment, a 3 mM stock was prepared from cisplatin powder (Millipore-Sigma, St. Louis, MO, USA, Cat#: 232120) biweekly in sterile water by vortexing and overnight incubation at 37 °C, with subsequent storage at RT while being protected from light. The stock was further diluted in media to various concentrations for treatment. Cultures were treated with cisplatin for 6 or 24 h, starting on day 6 in culture, for DNA damage and release/neurite outgrowth experiments, respectively.

### 4.3. Calcitonin Gene-Related Peptide Release

Release experiments were performed as described previously [7,34]. The wells were rinsed one time with HEPES buffer containing 25 mM HEPES, 135 mM NaCl, 3.5 mM KCl, 2.5 mM CaCl_2_, 1 mM MgCl_2_, 3.3 mM d-glucose, and 0.1% bovine serum albumin, pH 7.4. The cells were maintained at 37 °C for 3 incubations. During the first interval, cells were incubated with HEPES buffer for 20 min to establish resting basal release of immunoreactive CGRP (iCGRP), which we will refer to as CGRP. During the second interval, cells were incubated with HEPES containing 30 nM capsaicin in order to stimulate CGRP release. The third incubation was in HEPES to reestablish resting basal release. Supernatants were collected after every interval, and CGRP was measured using radioimmunoassay as previously described [7,34]. At the completion of the release experiment, cells were incubated for 20 min in 0.1 N HCl, and the supernatant was collected to determine total CGRP content. Release and total content of CGRP from each individual well of sensory neurons was considered an independent replicate; however, these cultures were derived from a minimum of 3 rats. 

### 4.4. Neurite Length Assessment

Plates were fixed with 4% paraformaldehyde for 20 min at RT and washed in PBS. Slides were blocked with 5% normal donkey serum for 1 h at RT and then washed 3 times in PBS. Chicken anti- PGP9.5 (1:250 dilution; Cat#: PA1-10011, Thermo Fisher Scientific, Waltham, MA, USA) antibody in 5% normal donkey serum and 0.05% Triton-X100 was added to the plate and it was incubated in a humidified chamber at 4 °C overnight. The wells were washed three times with PBS for 5 min each. Donkey anti-rabbit Alexa Fluor 647 conjugate (1:250 dilution; Cat#: 703-605-155, Jackson ImmunoResearch Labs, West Grove, PA, USA) was added to the plate for 2 h at RT in the dark. The wells were briefly washed 3 times using PBS. The plate was placed in the Cytation 5 Cell Imaging Multi-Mode Reader (BioTek Instruments, Winooski, VT, USA) and images were obtained using the Cy5 channel using the following parameters for cell bodies (min width of cell body: 25 μm, max width of cell body: 100 μm, detection threshold: 15,000 pixel intensity) and outgrowths (detection threshold: 10,000 pixel intensity, minimum cell growth to log as significant: 10 μm), modified from Pittman et al. [41]. 23–25 images each with an area of 394 × 291 μm were taken for each experimental condition, and the automated rolling ball algorithm was used to remove neuronal cell bodies and the remaining neurites were analyzed for Total Neurite Area measured in microns using Microsoft Excel (Microsoft Corporation, Redmond, WA, USA) and GraphPad Prism Version 7 (GraphPad Software, La Jolla, CA, USA). 

### 4.5. Staining and Quantification of 7,8-Dihydro-8-oxo-2′-deoxyguanosine (8-oxodG) and Cisplatin Adducts

Cultures, treated with vehicle or cisplatin (30 μM) for 6 h, were fixed for at least 20 min to overnight in −20 °C methanol stored at −20 °C, followed by −20 °C acetone for 15 min in −20 °C. Wells were then air-dried and washed 3 times in PBS for 5 min each. Cells were treated with 100 ug/mL RNase (Thermo-Fisher) in 150 mM sodium chloride, and 15mM sodium citrate incubated for 1 h at 37 °C. Slides were then washed sequentially for 3 min each with: PBS, 35% ethanol, 50% ethanol, and 75% ethanol at RT. Slides were then treated with 0.15N NaOH in 70% EtOH for 8 min at room temperature and then washed in PBS. Slides were then washed sequentially for 2 min each at RT with 4% paraformaldehyde in 70% EtOH, 50% ethanol, 35% ethanol, PBS. Slides were then incubated for 10 min with 5ug/mL proteinase K in 20 mM Tris, 1 mM EDTA at pH of 7.5 in 37 °C. Slides were washed 3 times in PBS at RT. Slides were blocked with 5% normal donkey serum for 1 h at RT and then washed 3 times in PBS. Mouse anti-8-oxodG (1:250 dilution; Cat#: 4354-MC-050, R&D Systems, Minneapolis, MN, USA), rabbit anti-cisplatin modified DNA [CP9/19] (1:500 dilution; Cat#: Ab00612-23, Absolute Antibody, Wilton, UK), and chicken anti-PGP9.5 (1:250 dilution; Cat#: PA1-10011, Thermo Fisher Scientific) antibodies in 1% BSA and 0.01% Tween-20 were added to the slides and incubated in a humidified chamber at 4 °C overnight. Slides were washed three times with PBS containing 0.05% Tween-20 for 5 min each. Donkey anti-Mouse Alexa Fluor 488 conjugate (Cat#: A21202, Thermo Fisher Scientific), donkey anti-Rabbit Alexa Fluor 568 conjugate (Cat#: A10042, Thermo Fisher Scientific), and donkey anti-chicken Alexa Fluor 647 (Cat#: 703-605-155, Jackson ImmunoResearch) secondary antibodies, all at 1:250 dilution, were added to the slides for 1 h at RT in the dark. Slides were briefly washed 3 times using PBS containing 0.05% Tween-20, and then with de-ionized water prior to removal of the gaskets per manufacturer’s instructions. Slides were coverslipped with VectaShield antifade mounting media containing DAPI (Cat#: H-1800-10, Vector Labs, Burlingame, CA, USA). Z-stack images were taken with the Cytation 5 Cell Imaging Multi-Mode Reader (BioTek Instruments) and GFP and TRITC channel measurements of integrated density were used for analysis of immunofluorescence images [76]. Cytation 5 automated image preprocessing and background flattening was carried out, followed by five iterations of deconvolution based on image objective. A DAPI mask was established using a threshold of 5000-pixel intensity and was applied to images for PGP9.5, where the PGP9.5 mask was established with a threshold of greater than or equal to 14,120-pixel intensity. The ROIs for 8-oxodG and DNA adducts were measured for integrated density relative to DAPI-integrated density within the mask for PGP9.5. Threshold levels were optimized by comparing ROIs generated to true neurons in the images and all parameters remained consistent for all images processed.

### 4.6. Reagents

All materials, unless stated otherwise, were purchased from Millipore-Sigma (St. Louis, MO, USA). F-12 media, horse serum, antibiotics, Nunc™ Lab-Tek™ II Chamber Slides™ (Cat#: 154526) and RNase (Cat #: EN0531) were acquired from Thermo Fisher Scientific. Paraformaldehyde (32% aqueous solution; Cat#: 15714S) was acquired from Electron Microscopy Sciences (Hatfield, PA, USA), the proteinase K (20 mg/mL; Cat#: 501-PK) from Viagen Biotech (Los Angeles, CA, USA), and the normal donkey serum (Cat#: 017-000-121) was acquired from Jackson ImmunoResearch (West Grove, PA, USA). TH5487 was acquired from Tocris (Cat#: 6749) and ARI3 from Millipore-Sigma (St. Louis, MO, USA; Cat#: 262017).

### 4.7. Statistical Analysis

Data were analyzed by one-way analysis of variance (ANOVA) or two-way ANOVA as indicated, and post hoc analyses were performed using Tukey’s or Dunnett’s test, as indicated. Statistical calculations were performed with the GraphPad Prism version 6.02 statistical software (GraphPad Software, La Jolla, CA, USA). Data are presented as mean ± SEM, and differences are considered significant if *p* < 0.05.

## Data Availability

The data presented in this study are available within the article.
